# Clinical Features and Implications of Albuminuria Trajectories in Type 2 Diabetes: The Fremantle Diabetes Study Phase 2

**DOI:** 10.1210/jendso/bvaf062

**Published:** 2025-04-08

**Authors:** Wendy A Davis, Aron Chakera, S A Paul Chubb, Timothy M E Davis

**Affiliations:** Medical School, University of Western Australia, Fremantle Hospital, Fremantle, WA 6160, Australia; Harry Perkins Institute for Medical Research, University of Western Australia, Crawley, WA 6009, Australia; Renal Unit, Sir Charles Gairdner Hospital, Nedlands, WA 6009, Australia; Medical School, University of Western Australia, Fremantle Hospital, Fremantle, WA 6160, Australia; Medical School, University of Western Australia, Fremantle Hospital, Fremantle, WA 6160, Australia; Department of Endocrinology and Diabetes, Fiona Stanley and Fremantle Hospitals Group, Murdoch, WA 6150, Australia

**Keywords:** type 2 diabetes, urinary albumin excretion, mortality, temporal trends, community-based, longitudinal study

## Abstract

**Context:**

The urinary albumin:creatinine ratio (uACR) can exhibit significant temporal changes but few studies have characterized transition patterns between uACR categories in type 2 diabetes.

**Objective:**

The study aim was to use group-based trajectory modeling (GBTM) to identify clusters of people with type 2 diabetes and distinct uACR trajectories.

**Methods:**

Of 1482 participants in the observational Fremantle Diabetes Study Phase 2, a total of 1145 (77.3%; mean age 65.4 years, 53.3% males) with 2 or more biennial uACR measurements over 6 years were included in GBTM. Independent baseline associates of uACR trajectory group membership were assessed using multinomial regression. Associations between group membership and changes in estimated glomerular filtration rate over 4 years were explored.

**Results:**

The optimum GBTM model comprised 6 categories: normoalbuminuria (n = 429, 37.5%), regression (n = 82, 7.2%), progression (n = 71, 6.2%), progression/regression (n = 104, 9.1%), persistent microalbuminuria (n = 401, 35.0%), and persistent macroalbuminuria (n = 58, 5.1%). The latter 5 groups had worse glycemic control than the normoalbuminuria group. The 3 groups starting from/returning to normoalbuminuria had heterogeneous baseline characteristics but a decline in renal function that was similar to the normoalbuminuric group. The persistent microalbuminuria group had adverse baseline cardiometabolic features and longitudinal renal outcomes relative to the normoalbuminuria/other microalbuminuria groups. The persistent macroalbuminuria group had, consistent with its baseline characteristics, the highest mortality (31.0% vs ≤18.5% in the other groups) and most rapid progression of renal dysfunction.

**Conclusion:**

GBTM identified distinct uACR trajectory groups with clinical and prognostic implications, and could be used to stratify participants in clinical trials of new therapies for diabetic kidney disease.

Although the urinary microalbumin excretion rate (uAER) has long been recognized as a valuable marker of early diabetic kidney disease (DKD) [1], it exhibits significant intraindividual day-to-day variability [2-5]. Timed urine collections required for measurement of uAER are demanding and often inaccurate, and so a spot first-morning urine albumin:creatinine ratio (uACR) has been adopted as a pragmatic alternative [6, 7]. However, uACR also has a high degree of intraindividual variability [8-10] and so serial (at least annual) measurements are recommended for establishing and monitoring the progression or regression of DKD alongside the estimated glomerular filtration rate (eGFR) [10].

There have been attempts to categorize serial changes both in uAER and uACR. Initial studies used subjective classification of the temporal patterns of uAER within an individual as persistent, intermittent, progressing or (except for normoalbuminuria) regressing, with or without further initial grouping by baseline normoalbuminuria, microalbuminuria, or macroalbuminuria [4, 11], thus generating up to 11 subgroups of participants [4]. Subsequent studies have employed trajectory modeling as an objective alternative. In one study of clinic-based Chinese people with type 2 diabetes using this approach to assess relationships between temporal changes in uACR and cardiovascular outcomes, 4 groups were identified, namely low-stable, moderate-stable, high-stable group, and elevated-increasing, without a category for uACR regression [12]. A study of progression to end-stage kidney disease or death in people with type 2 diabetes and biopsy-proven DKD found 3 trajectories, high-increasing, high-decreasing, and low-stable [13]. In 2 larger general population studies of cardiovascular outcomes, latent class modeling generated 5 (low-stable, moderate-stable, high-stable group, moderate-increasing, and high-increasing) [14] and 8 (1 regressing group and 7 stable/increasing) [15] uACR trajectory groups, respectively.

Given the heterogeneity between published studies in terms of participant selection, length of follow-up and statistical methods, the aim of the present study was to use group-based trajectory modeling (GBTM) to identify clusters of people exhibiting distinct uACR trajectories in a well-characterized, representative community-based cohort of people with type 2 diabetes followed for 6 years. We also related group membership to contemporaneous serial changes in eGFR.

## Materials and Methods

### Study Site, Participants, and Approvals

**Figure 1.**
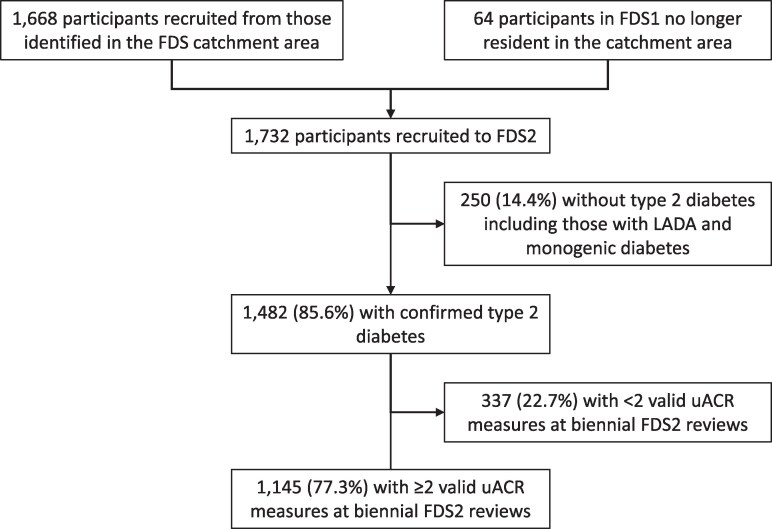
Consort diagram showing the Fremantle Diabetes Study Phase 2 participants involved in the present study.

The Fremantle Diabetes Study Phase 2 (FDS2) is an observational cohort study conducted in a zip code–defined urban community of 157 000 people surrounding the port city of Fremantle in the state of Western Australia (WA) [16]. Socioeconomic data relating to income, employment, housing, transportation, and other variables in the study area show an average Index of Relative Socio-economic Advantage and Disadvantage of 1033 with a range by zip code of 977-1113, figures similar to the Australian national mean ± SD which are set at 1000 ± 100 [17]. Descriptions of FDS2 recruitment, sample characteristics, and details of identified but nonrecruited people with diabetes have been published [16]. Individuals resident in the catchment area with a clinician-verified diagnosis of diabetes (excluding gestational diabetes) were identified through all available hospital and community sources. Of 4639 with known diabetes found between the years 2008 and 2011, a total of 1668 (36.0%) were recruited to FDS2. Sixty-four former FDS phase 1 participants recruited between 1993 and 1996 who had moved out of the catchment area were also recruited, giving a total cohort of 1732. For the purposes of the present study, we included those FDS2 participants with type 2 diabetes who did not have monogenic diabetes or latent autoimmune diabetes of adults after relevant screening tests [17] (see Fig. 1).

### Study Procedures

All FDS2 participants were invited to face-to-face assessments at entry and then biennially, interspersed with biennial postal questionnaires [16]. Face-to-face assessments included a standardized comprehensive questionnaire and physical examination, and fasting biochemical tests performed in a single nationally accredited laboratory. Participants were requested to bring all medications and/or prescriptions to each visit and details were verified and recorded. Racial/ethnic background was categorized based on self-selection, country/countries of birth and parents’/grandparents’ birth, and language(s) spoken at home as Anglo-Celt, Southern European, Other European, Asian, Aboriginal or mixed/other. Smoking, alcohol consumption and vaccination histories were documented. Body mass index (BMI) was determined together with a body shape index (ABSI), which represents a more reliable estimate of visceral adiposity [18].

Complications of diabetes were identified using standard definitions [19]. Albuminuria was assessed by early-morning spot uACR measurement and renal impairment from the eGFR [20]. Sex-specific cutoffs for microalbuminuria and macroalbuminuria were used, namely normoalbuminuria less than 2.5 and less than 3.5 mg/mmol, microalbuminuria 2.5 to 25 and 3.5 to 35 mg/mmol, and macroalbuminuria greater than 25 and greater than 35 mg/mmol for men and women, respectively [21]. Distal symmetrical polyneuropathy (DSPN) was defined using the vibration perception threshold [22]. Retinopathy was defined as one microaneurysm in either eye or worse and/or previous laser treatment on fundus photography and/or specialist ophthalmological assessment. Participants were classified as having coronary heart disease if there was a history of myocardial infarction, angina, coronary artery bypass grafting, or angioplasty, and as having cerebrovascular disease if there was a history of stroke and/or transient ischemic attack. Peripheral arterial disease (PAD) was defined as an ankle brachial index less than or equal to 0.90 or a diabetes-related lower-extremity amputation.

The Hospital Morbidity Data Collection (HMDC) contains validated information regarding all public/private hospitalizations in WA since 1970, and the Death Register contains information on all deaths in WA [23]. The FDS2 database has been linked to these databases through the WA Data Linkage System (WADLS), as approved by the WA Department of Health Human Research Ethics Committee. The HMDC was used to supplement data obtained through FDS assessments relating to prevalent/prior complications and conditions during the 5 years prior to study entry. These data were used to calculate the Charlson Comorbidity Index (CCI) [24] excluding diabetes-specific chronic complications [25].

### Statistical Analysis

The computer packages IBM SPSS Statistics 29 (IBM Corporation) and StataSE 15 (StataCorp LP) were used for statistical analysis. Data from the baseline (year 0), year 2, year 4, and year 6 assessments were used. Data are presented as proportions, mean ± SD, geometric mean (SD range), or, in the case of variables that did not conform to an approximately normal or log-normal distribution, median and interquartile range (IQR). For independent samples, 2-sample comparisons were by Fisher's exact test for proportions, *t* test for normally distributed variables, and Mann-Whitney *U* test for nonparametric variables. Comparisons between multiple groups for categorical variables were by the Fisher-Freeman-Halton exact or chi-square tests, for normally or log-normally distributed continuous variables by one-way analysis of variance, and for variables not conforming to normal or log-normal distribution by Kruskal-Wallis test. Where the overall trend for these multiple comparisons was statistically significant, post hoc Bonferroni-corrected pairwise comparisons were performed. A 2-tailed significance level of *P* less than .05 was used throughout.

### Trajectory Group Selection

GBTM identifies distinct groups of individuals following similar progressions of an observable measure over time. The group-based approach is an example of a finite mixture model that assumes the presence of unobserved groups, called latent classes, within an overall population. Maximum likelihood is used to estimate model parameters. The equation describing the likelihood of an individual's observed repeated measures is composed of two elements, the probability of group membership and the probability of the observed data given group membership. The probability of group membership is modeled with a generalized logit model.

GBTM was used to identify albuminuria category trajectory groups. Since management strategies and their intensity are based, in part, on categories of albuminuria [26], censored normal models were used to estimate trajectories within and between albuminuria categories over 6 years (4 biennial assessments). To assist model selection, the Bayesian Information Criterion (BIC) was used to determine the optimum number of groups and their functional form (flat, linear, quadratic, or cubic) [27]. BIC values balance model fit with model complexity, and the closer the negative BIC value is to zero, the better the fit. Other selection criteria included i) Bayes Index greater than 10 (very strong evidence that the preferred model is better than the alternative model), ii) adequate numbers of participants in each group, iii) distinct trajectories, iv) narrow CIs, v) average posterior probabilities of group membership greater than 0.7 for each group, vi) odds of correct classification based on posterior probabilities of group membership greater than 5 for each group, vii) close correspondence between each group's estimated probability and the proportion of participants classified to that group according to the maximum posterior probability assignment rule, and viii) models in which the variance matrix was not nonsymmetric or highly singular. To help guide selection and aid interpretation, plots of the individual trajectories for each trajectory group were also generated.

### Characteristics of Trajectory Groups

The bivariable characteristics of the trajectory groups were determined and multinomial regression used to identify independent associates of group membership. Clinically relevant and biologically plausible variables were considered for model entry if bivariable *P* was less than .20. Loss to follow-up, defined as no valid uACR measure at year 6, was quantified by trajectory group and a logistic regression analysis undertaken to identify associates of dropout. If the magnitude of dropout differed by trajectory group, it was adjusted for in the final multinomial models. Binomial logistic regression analyses were undertaken where warranted to further investigate differences in the characteristics of neighboring trajectory groups.

## Results

### Sample Characteristics

Of 1482 participants with confirmed type 2 diabetes, 1145 (77.3%) had at least 2 valid uACR measures with the median (IQR) number being 4 (3-4), 4 being the maximum number possible. The 337 with fewer than 2 valid uACR measures were not significantly older (66.8 ± 12.9 vs 65.4 ± 11.1 years; *P* = .073), but were more likely to be female (54.3% vs 46.7%; *P* = .016), had diabetes diagnosed longer (11.0 [4.0-18.0] vs 8.0 [2.3-15.3] years; *P* < .001) and higher uACR (4.0 [0.9-18.3] vs 3.2 [0.9-11.7]; *P* = .019) compared to those with 2 or more uACR measures.

### Trajectory Group Selection and Evaluation

**Figure 2.**
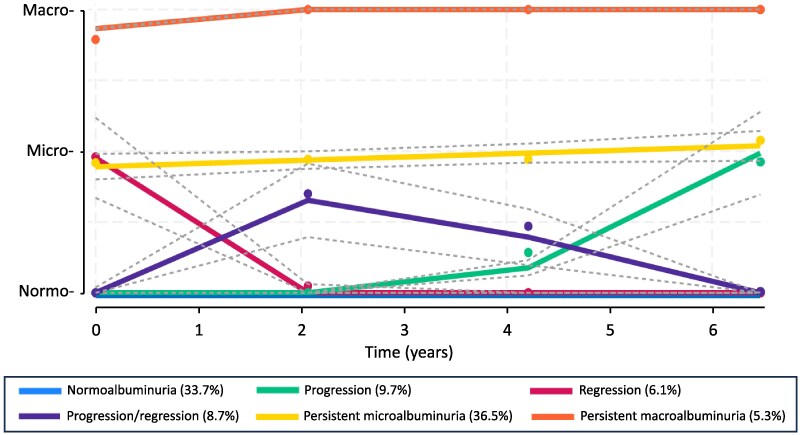
Six predicted albuminuria trajectory groups derived from group-based trajectory modelling which best fitted the serial data. 95% CIs are shown as dotted lines.

**Figure 3.**
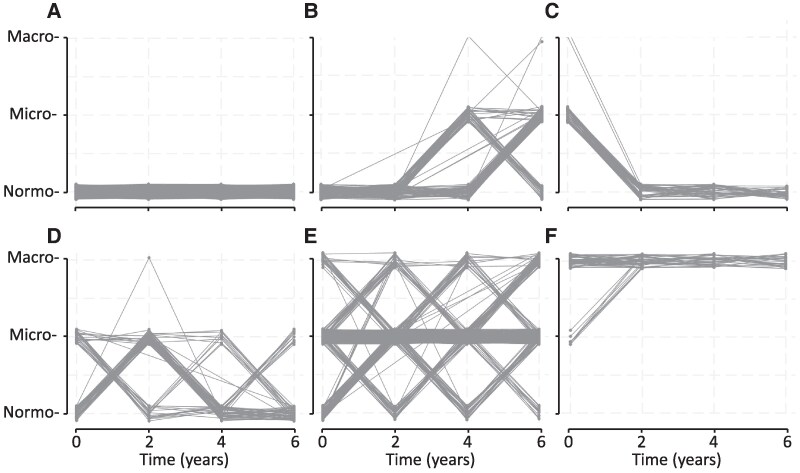
Individual plots of uACR category data in the 6 trajectory groups. A, normoalbuminuria; B, progression; C, regression; D, progression/regression; E, persistent microalbuminuria; F, persistent macroalbuminuria.

Taking all selection criteria into consideration, the best model for the albuminuria categories data was one with 6 groups. Changes in albuminuria category and CIs for the 6 trajectory groups over time are shown in Fig. 2. Plots of the individual trajectories for each trajectory group are presented in Fig. 3 for the 1144 individuals (99.9%) with uACR measured at the baseline assessment and at least 1 of the scheduled 2-year assessments. One group had a stable horizontal trajectory at the normoalbuminuric level (n = 429 [38%], “normoalbuminuria”), 1 started with normoalbuminuria but progressed to microalbuminuria after the second year of follow-up but not to macroalbuminuria by 6 years (n = 82 [7%], “progression”), 1 started with microalbuminuria but regressed toward stable normoalbuminuria within the first 2 years of follow-up (n = 71 [6%], “regression”), 1 progressed toward microalbuminuria but then regressed back toward normoalbuminuria after 2 years in most individuals (104 [9%], “progression/regression”), 1 had microalbuminuria with an overall slight upward trajectory but with the most variability within individual participants (n = 401 [35%], “persistent microalbuminuria”), and 1 had persistent macroalbuminuria after a slightly upward trajectory in the first 2 years (n = 58 [5%]).

The average posterior probabilities for the trajectory groups were 0.90 (normoalbuminuria), 0.95 (progression), 0.90 (regression), 0.84 (progression/regression, 0.98 (persistent microalbuminuria), and 0.98 (persistent macroalbuminuria), all greater than the recommended cutoff of greater than 0.7. The odds of correct classification based on the posterior probabilities of group membership were greater than 14 for all groups (Table 1).

**Table 1.** Proportion of study participants classified to each group according to the estimated probability and the maximum posterior probability assignment rule, average posterior probability and odds of correct classification for albuminuria trajectory groups

	Normoalbuminuria	Progression	Regression	Progression/ regression	Persistent microalbuminuria	Persistent macroalbuminuria
p_e_ (%)	33.7	9.7	6.1	8.7	36.5	5.3
p (%)	37.5	7.2	6.2	9.1	35.0	5.1
Average posterior probability (app)	0.90	0.95	0.90	0.84	0.98	0.98
Odds of correct classification (occ)*^a^*	14.9	238.3	130.5	53.2	97.9	1012.7
Odds of correct classification using weighted posterior probabilities (occ_w_)*^b^*	17.6	171.1	132.8	55.8	91.8	965.5

p_e_ = estimated group probability based on sums of posterior probabilities; p = number in trajectory group/total number.

^a^occ = n/d where n = app/(1-app) and d = p/(1-p).

^b^occ_w_ = n/d_w_ where d_w_ = p_e_/(1-p_e_).

### Attrition Within Trajectory Groups

Attrition during follow-up, defined as no valid year 6 uACR measurement (Table 2), was highly significant by trajectory group (*P* < .001). Forty percent of dropouts were explained by deaths during study follow-up, which were also strongly trajectory group dependent (*P* < .001). Attrition was independently associated with baseline age, Aboriginal background, lack of English fluency, currently married/de facto relationship (inversely), current smoker, heart rate, uACR, eGFR less than 60 mL/min/1.73 m^2^, DSPN, and PAD. Mortality over the 6 years of follow-up was highest in the persistent macroalbuminuria group (31.0%) compared with less than or equal to 18.5% in the other trajectory groups (see Table 2).

**Table 2.** Numbers of participants and cumulative deaths at each assessment in the albuminuria trajectory groups

	All	Normoalbuminuria	Progression	Regression	Progression/ regression	Persistent microalbuminuria	Persistent macroalbuminuria
Total No. (%) per group	1145	429 (37.5)	82 (7.2)	71 (6.2)	104 (9.1)	401 (35.0)	58 (5.1)
N (%) assessed at baseline	1144 (99.9)	428 (99.8)	82 (100.0)	71 (100.0)	104 (100)	401 (100.0)	58 (100.0)
N (%) assessed at y 2	1134 (99.0)	426 (99.3)	82 100.0)	70 (98.6)	103 (99.0)	397 (99.0)	56 (96.7)
N (%) assessed at y 4	868 (75.8)	325 (75.8)	79 (96.3)	47 (66.2)	84 (80.8)	301 (75.1)	32 (55.2)
N (%) assessed at y 6	735 (64.2)	297 (69.2)	71 (86.6)	35 (49.3)	58 (55.8)	249 (62.1)	25 (43.1)
N (%) deaths by end y 2 follow-up*^a^*	58 (5.1)	15 (3.5)	0 (0)	4 (5.6)	4 (3.8)	28 (7.0)	7 (12.1)
N (%) deaths by end y 4 follow-up*^b^*	102 (8.9)	25 (5.8)	2 (2.4)	9 (12.7)	11 (10.6)	43 (10.7)	12 (20.7)
N (%) deaths by end y 6 follow-up*^c^*	163 (14.2)	35 (8.2)	6 (7.3)	12 (16.9)	18 (17.3)	74 (18.5)	18 (31.0)
Drop-out before y 6	410 (35.8)	132 (30.8)	11 (13.4)	36 (50.7)	46 (44.2)	152 (37.9)	33 (56.9)

Abbreviation: uACR, urinary albumin:creatinine ratio.

^a^All had a valid year 2 uACR measurement.

^b^Valid year 4 uACR measurement was conducted in 3, 2, 3, 8, 9, and 4 participants in the normoalbuminuria, progression, regression, progression/regression, persistent microalbuminuria, and persistent macroalbuminuria trajectory groups, respectively, before death occurred prior to year 4 closeout.

^c^Valid year 6 uACR measurement was conducted in 1, 2, 0, 2, 9, and 2 participants in the normoalbuminuria, progression, regression, progression/regression, persistent microalbuminuria, and persistent macroalbuminuria trajectory groups, respectively, before death occurred prior to year 6 closeout.

### Characteristics of the Trajectory Groups

The baseline characteristics of the 6 trajectory groups at baseline are summarized in Table 3. There was a trend toward increasing age, longer diabetes duration, worse glycemic control despite greater insulin use, and greater macrovascular disease burden across the groups. In more detail, those in the persistent macroalbuminuria group had i) significantly higher systolic and diastolic blood pressures, and a lower eGFR, than all other groups; ii) higher serum triglycerides than all groups other than the regression group; iii) a higher ABSI and diabetes duration, and a higher proportion with comorbidities, than those in the normoalbuminuria, progression, regression, and progression/regression trajectory groups; iv) a higher glycated hemoglobin A_1c_ (HbA_1c_) than those in the normal, progression, and progression/regression groups; v) a higher percentage of current smokers than the normoalbuminuria, progression/regression, and persistent microalbuminuria groups; vi) a higher proportion of Aboriginal Australians compared with the normoalbuminuria, progression, and persistent microalbuminuria groups; and vii) a higher fasting glucose and a higher percentage of participants with retinopathy and cerebrovascular disease than those in the normoalbuminuria and progression/regression groups. The persistent microalbuminuria trajectory group had i) a longer diabetes duration than all but the persistent macroalbuminuria group, ii) a higher systolic blood pressure and greater percentage with ischemic heart disease than the normoalbuminuria and progression groups, iii) a higher ABSI and a greater proportion of men than the normoalbuminuria and regression groups, and were more likely to be taking antihypertensive medications and to have cerebrovascular disease than those in the normal and progression/regression groups.

**Table 3.** Baseline characteristics categorized by albuminuria trajectory group

	Normoalbuminuria	Progression	Regression	Progression/ regression	Persistent microalbuminuria	Persistent macroalbuminuria	*P*
No. (%)	429 (37.5)	82 (7.2)	71 (6.2)	104 (9.1)	401 (35.0)	58 (5.1)	
uACR, mg/mmol	1.2 (0.8-2.0)	1.5 (1.0-2.2)	5.8 (3.0-11.2)*^c^*^,*f*^	1.6 (1.0-2.4)*^c^*^,*h*^	7.0 (2.8-18^)*c,f*,*i,l*^	73 (22-248)^*c*,*f*,*i*,*l*,*o*^	<.001
Age, y	62.7 ± 10.3	64.9 ± 10.5	65.2 ± 11.7	65.7 ± 11.2	68.2 ± 10.7*^c^*	67.0 ± 14.4	<.001
Age at diabetes diagnosis, y	55.5 ± 10.8	56.3 ± 12.4	57.3 ± 10.9	56.4 ± 12.1	56.1 ± 11.9	51.7 ± 14.2	.098
Diabetes duration, y	5.0 (1.3-12.1)	5.0 (1.2-15.4)	6.6 (2.0-13.3)	7.5 (2.4-15.0)	12.0 (6.0-16.9)*^c^*^,*e*,*h*,*k*^	14.6 (4.0-23.4)*^c^*^,*e*,*h*,*j*^	<.001
Sex, % male	48.0	52.4	39.4	52.9	59.6*^a^*^,*g*^	67.2*^a^*	<.001
Ethnic background, %						*^b^*	.001
Anglo-Celt	60.1	53.7	52.1	54.8	56.4	44.8	
Southern European	8.6	11.0	12.7	12.5	13.7	15.5	
Other European	6.3	8.5	5.6	8.7	8.0	3.4	
Asian	4.0	2.4	5.6	1.9	4.7	6.9	
Aboriginal	3.5	2.4	11.3	4.8	5.0	19.0	
Mixed/other	17.5	22.0	12.7	17.3	12.2	10.3	
Not fluent in English, %	5.6	3.7	14.1	15.4*^a^*	10.0	12.1	.002
Education after primary level, %	92.6	92.6	83.8	89.4	86.8	82.8	.019
Married/de facto relationship, %	66.9	70.7	54.9	62.5	63.6	48.3	.036
Smoking status, %						*^b,j^*	.003
Never	45.3	50.0	47.9	43.3	36.8	29.3	
Ex-smoker	47.7	37.8	45.1	51.9	53.8	46.6	
Current smoker	7.0	12.2	7.0	4.8	9.5	24.1	
Alcohol use, standard drinks/d	0.1 (0-1.2)	0.3 (0-1.5)	0.1 (0-0.8)	0.1 (0-0.8)	0.1 (0-1.5)	0.1 (0-1.5)	.360
BMI, kg m^−2^	31.4 ± 5.9	31.4 ± 5.8	31.4 ± 6.6	31.8 ± 5.1	31.2 ± 6.0	30.5 ± 5.5	.855
ABSI, m^11/6^ kg^−2/3^	0.080 ± 0.005	0.081 ± 0.004	0.080 ± 0.005	0.081 ± 0.006	0.082 ± 0.004*^c^*^,*g*^	0.084 ± 0.004*^c^*^,*e*,*h*,*j*^	<.001
Fasting serum glucose, mmol/L	6.8 (6.0-8.2)	7.0 (6.2-8.5)	7.2 (6.4-10.0)	7.1 (6.0-8.1)	7.4 (6.3-9.1)*^c^*	8.1 (6.8-11.4)*^c^*^,*j*^	<.001
HbA_1c_, %	6.6 (6.0-7.3)	6.7 (6.2-7.5)	7.0 (6.3-8.4)*^a^*	6.8 (6.2-7.5)	6.9 (6.4-7.9)*^c^*	7.5 (6.7-9.3)*^c^*^,*d,j*^	<.001
HbA_1c_, mmol/mol	49 (42-56)	50 (44-58)	53 (45-68)	51 (44-58)	52 (46-63)*^c^*	58 (50-78)*^c^*^,*d*,*j*^	<.001
Diabetes treatment, %					*^c^*	*^c^* ^,*d*^	<.001
Diet	32.2	31.7	22.9	26.9	18.7	6.9	
Noninsulin medications	53.4	50.0	62.9	52.9	52.9	56.9	
Insulin alone	3.0	4.9	1.4	3.8	6.2	10.3	
Insulin ± other agents	11.4	13.4	12.9	16.3	22.2	25.9	
Heart rate, beats/min	68 ± 11	69 ± 12	68 ± 12	68 ± 12	70 ± 13	73 ± 13*^a^*	.008
Systolic blood pressure, mm Hg	141 ± 19	140 ± 17	149 ± 20*^a^*	146 ± 19	149 ± 23*^c^*^,*e*^	162 ± 26*^c^*^,*f*,*h*,*l*,*o*^	<.001
Diastolic blood pressure, mm Hg	80 ± 11	80 ± 11	80 ± 11	80 ± 11	80 ± 13	86 ± 15*^b^*^,*d*,*h*,*j*,*n*^	.012
Antihypertensive therapy, %	64.1	75.6	72.5	72.1	86.0*^c^*^,*j*^	86.2*^b^*	<.001
Renin-angiotensin blocker therapy, %	55.2	74.4*^a^*	62.0	67.3	75.1*^c^*	77.6*^a^*	<.001
Total serum cholesterol, mmol/L	4.3 ± 1.0	4.5 ± 1.1	4.6 ± 1.1	4.3 ± 1.0	4.3 ± 1.2	4.5 ± 1.4	.294
Serum HDL-cholesterol, mmol/L	1.27 ± 0.35	1.22 ± 0.28	1.26 ± 0.37	1.23 ± 0.26	1.20 ± 0.30	1.20 ± 0.35	.067
Serum triglycerides, mmol/L	1.4 (0.9-2.2)	1.6 (1.0-2.4)	1.7 (1.0-2.8)	1.4 (0.9-2.1)	1.6 (0.9-2.8)*^c^*	2.0 (1.2-3.6)*^c^*^,*d*,*l,m*^	<.001
Lipid-modifying medication, %	68.3	70.7	59.4	71.2	73.8	81.0	.071
Aspirin use, %	32.9	42.7	31.9	41.7	42.9*^a^*	37.9	.045
eGFR, mL/min/1.73 m^2^	85.6 ± 16.6	82.7 ± 15.9	80.7 ± 19.0	82.1 ± 17.5	77.1 ± 20.9*^c^*	66.2 ± 30.8*^c^*^,*f*,*i*,*l,o*^	<.001
eGFR categories, %					*^c^*	*^c^* ^,*f*,*g,l,^o^*^	<.001
≥90 mL/min/1.73 m^2^	46.1	36.6	37.1	42.3	30.7	34.5	
60-89 mL/min/1.73 m^2^	45.9	54.9	47.1	45.2	49.5	24.1	
45-59 mL/min/1.73 m^2^	6.1	3.7	12.9	8.7	9.5	13.8	
30-44 mL/min/1.73 m^2^	1.9	4.9	1.4	3.8	8.3	13.8	
<30 mL/min/1.73 m^2^	0	0	1.4	0	2.0	13.8	
Any retinopathy, %	26.3	36.6	36.6	31.4	43.9*^c^*	59.6*^c^*^,*j*^	<.001
DSPN, %	29.7	40.2	31.4	33.7	44.6*^c^*	53.4*^b^*	<.001
PAD, %	14.7	18.3	22.5	22.1	23.8*^a^*	36.2*^b^*	<.001
Coronary heart disease, %	19.1	17.1	25.4	32.7	34.4*^c^*^,*d*^	43.1*^b^*^,*d*^	<.001
Cerebrovascular disease, %	4.0	3.7	7.0	2.9	12.2*^c^*^,*j*^	17.2*^b^*^,*j*^	<.001
CCI, %					*^c^*	*^c^* ^,*f*,*g*,*l*^	<.001
0	84.1	86.6	78.9	82.7	70.6	53.4	
1 or 2	12.1	9.8	18.3	14.4	20.4	27.6	
≥3	3.7	3.7	2.8	2.9	9.0	19.0	

Data are presented as percentages (%), mean ± SD, medians (interquartile range), geometric mean (SD range).

Abbreviations: ABSI, a body shape index; BMI, body mass index; CCI, Charlson Comorbidity Index; DSPN, distal symmetrical polyneuropathy; eGFR, estimated glomerular filtration rate; HbA_1c_, glycated hemoglobin A_1c_; HDL, high-density lipoprotein; PAD, peripheral arterial disease; uACR, urinary albumin:creatinine ratio.

Pairwise comparisons are Bonferroni-corrected: *^a^P* less than .05. *^b^P* less than .01. *^c^P* less than .001 vs normoalbuminuria. *^d^P* less than .05. *^e^P* less than .01. *^f^P* less than .001 vs progression. *^g^P* less than .05. *^h^P* less than .01. *^i^P* less than .001 vs regression. *^j^P* less than .05. *^k^P* less than .01. *^l^P* less than .001 vs progression/regression. *^m^P* less than .05. *^n^P* less than .01. *^o^P* less than .001 vs persistent microalbuminuria.

The results of multinomial regression analysis are summarized in Table 4. The models were adjusted for dropouts given the independent association with group membership. The predictors of albumin category trajectory group membership were the following:

- Demographic and behavioural variables—compared to the normoalbuminuria group, an increase of 10 years in age increased the odds of being in the progression and persistent microalbuminuria groups by 39% to 52%. Men were 1.5 and 2.6 times as likely as women to be in the persistent microalbuminuria and persistent macroalbuminuria groups, respectively. Participants not fluent in English had 2.6 to 2.7 times higher odds of being in the regression and progressing/regression groups. Current smokers had twice the odds of being in the progression and persistent microalbuminuria groups, and nearly 7 times the odds of being in the persistent macroalbuminuria group.
- Clinical variables—compared to the normoalbuminuria group, an increase of 5 years in the duration of diabetes increased the odds of being in the persistent microalbuminuria and persistent macroalbuminuria groups by 22% and 37%, respectively. An increase of 10 mm Hg in systolic blood pressure increased the odds of being in the regression, persistent microalbuminuria, and persistent macroalbuminuria groups by 20%, 14%, and 44%, respectively. The use of renin-angiotensin system (RAS)-blocking drugs was associated with more than double the odds of progression, persistent microalbuminuria, and persistent macroalbuminuria group membership. Cerebrovascular disease was associated with 2 to 3 times the odds of persistent microalbuminuria and persistent macroalbuminuria group membership.
- Laboratory variables—compared to the normoalbuminuria group, an increase of 1% (11 mmol/mol) in HbA_1c_ increased the odds of being in the progression, regression, progression/regression, persistent microalbuminuria, and persistent macroalbuminuria group by 27%, 36%, 21%, 32%, and 59%, respectively. An eGFR <45 mL/min/1.73 m^2^ was associated with 3 and 11 times the odds of persistent microalbuminuria and persistent macroalbuminuria group membership, respectively. Ln(serum triglycerides) was associated with 2- to 4-fold increased odds of group membership across all groups except progression/regression.

**Table 4.** Multinomial logistic regression model of influential baseline factors on albuminuria category predicted trajectory membership relative to the normoalbuminuria group in type 2 diabetes, adjusted for attrition

	Progression	Regression	Progression/regression	Persistent microalbuminuria	Persistent macroalbuminuria
Age (increase of 10 y)	**1.39 (1.06-1.82)**	1.29 (0.98-1.69)	1.17 (0.93-1.48)	**1.52 (1.29-1.80)**	1.37 (0.96-1.95)
Male	1.14 (0.70-1.87)	0.71 (0.42-1.20)	1.16 (0.75-1.81)	**1.52 (1.12-2.07)**	**2.63 (1.35-5.14)**
Not fluent in English	0.79 (0.23-2.75)	**2.55 (1.12-5.79)**	**2.69 (1.34-5.41)**	1.73 (0.97-3.10)	2.07 (0.74-5.79)
Current smoker	**2.28 (1.01-5.15)**	0.97 (0.34-2.79)	0.72 (0.26-1.97)	**2.01 (1.14-3.54)**	**6.65 (2.70-16.4)**
Diabetes duration (increase of 5 y)	1.03 (0.86-1.23)	0.91 (0.75-1.12)	1.07 (0.91-1.25)	**1.22 (1.09-1.36)**	**1.37 (1.13-1.66)**
HbA_1c_ (increase of 1% or 11 mmol/mol)	**1.27 (1.04-1.55)**	**1.36 (1.13-1.63)**	**1.21 (1.01-1.44)**	**1.32 (1.17-1.50)**	**1.59 (1.30-1.95)**
Systolic blood pressure (increase of 10 mm Hg)	0.95 (0.83-1.09)	**1.20 (1.05-1.35)**	1.09 (0.98-1.22)	**1.14 (1.05-1.23)**	**1.44 (1.25-1.65)**
On ACE-I/ARB	**2.36 (1.35-4.11)**	1.40 (0.80-2.43)	1.54 (0.96-2.46)	**2.04 (1.47-2.85)**	**2.91 (1.37-6.20)**
Ln(serum triglycerides (mmol/L))*^a^*	**1.94 (1.13-3.33)**	**2.12 (1.23-3.66)**	1.09 (0.67-1.76)	**2.26 (1.62-3.15)**	**4.11 (2.20-7.69)**
eGFR <45 mL/min/1.73 m^2^	2.50 (0.67-9.27)	0.94 (0.19-4.76)	1.44 (0.40-5.11)	**3.02 (1.31-6.95)**	**11.1 (3.82-32.3)**
Cerebrovascular disease	0.83 (0.23-3.00)	1.81 (0.61-5.35)	0.60 (0.17-2.14)	**2.46 (1.30-4.66)**	**3.16 (1.16-8.65)**

N = 1132/1145 (99%) in final model. Data presented are odds ratio (95% CI). Bold type denotes statistically significant result (*P* < .050). Goodness of fit: Pearson chi-square 5374.8, degree of freedom = 5595, *P* = .982. Nagelkerke pseudo R^2^: 0.322.

Abbreviations: ACE-I, angiotensin-converting enzyme I; ARB, angiotensin receptor blocker; eGFR, estimated glomerular filtration rate; HbA_1c_, glycated hemoglobin A_1c_.

^a^An increase of 1 in ln(serum triglycerides) equates to an increase of 2.72 in serum triglycerides.

### Relationship Between Albuminuria Trajectory Group and Changes in Renal Function

To maximize participant inclusion in light of attrition and to capture clinically meaningful changes in eGFR over time [28], 4-year changes in eGFR (absolute and percentage) by predicted albuminuria trajectory group were analyzed. These are summarized in Table 5. Overall there was a highly significant increasing trend in each metric of eGFR change during follow-up (all *P* < .001). Those in the persistent macroalbuminuria group had a significantly higher percentage change in eGFR between baseline and year 4 both overall and per year vs all other predicted trajectory groups (all *P* < .001). One-third of those in the persistent macroalbuminuria group had a change in eGFR greater than 30% between baseline and year 4, a significantly greater percentage than all but the relatively small regression group.

**Table 5.** Four-year changes in estimated glomerular filtration rate by albuminuria predicted trajectory

	Normal	Progression	Regression	Progression/regression	Persistent microalbuminuria	Persistent macroalbuminuria	*P*
No. (%)	324 (36.9)	80 (9.1)	47 (5.4)	85 (9.7)	308 (35.1)	33 (3.8)	
Change in eGFR between baseline and y 4, mL/min/1.73 m^2^	−6 ± 9	−5 ± 9	−9 ± 12	−7 ± 9	−8 ± 11	−13 ± 12*^b,d,g^*	<.001
Annual change in eGFR between baseline and y 4, mL/min/1.73 m^2^ per y	−1.5 ± 2.2	−1.2 ± 2.2	−2.1 ± 3.0	−1.7 ± 2.1	−1.9 ± 2.6	−3.4 ± 3.6*^c^*^,*e*,*h*,*j*^	<.001
% change in eGFR between baseline and y 4, %	−7 ± 12	−6 ± 12	−11 ± 17	−8 ± 13	−11 ± 16*^a^*	−24 ± 21*^c^*^,*e*,*f*,*i*,*k*^	<.001
Annual % change in eGFR between baseline and y 4, % per y	−1.6 ± 2.8	−1.5 ± 2.8	−2.5 ± 3.9	−2.1 ± 3.0	−2.5 ± 3.8*^a^*	−6.1 ± 6.0*^c^*^,*e*,*f*,*i*,*k*^	<.001
Change in eGFR between baseline and y 4 > 30% (%)	3.1	1.3	10.6	5.9	11.4*^c^*	33.3*^c,e,h,j^*	<.001

Data are presented as percentages (%), mean ± SD, medians (interquartile range), geometric mean (SD range).

Abbreviation: eGFR, estimated glomerular filtration rate.

Pairwise comparisons are Bonferroni-corrected:

^*a*^ *P*<.05.

^*b*^ *P*<.01.

^*c*^ *P*<.001 vs normoalbuminuria.

^*d*^ *P*<.01.

^*e*^ *P*<.001 vs progression.

^*f*^ *P*<.001 vs regression.

^*g*^ *P*<.05.

^*h*^ *P*<.01.

^*i*^ *P*<.001 vs progression/regression.

^*j*^ *P*<.05.

^*k*^ *P*<.001 vs persistent microalbuminuria.

## Discussion

The recommended regular (at least annual [10]) measurement of uACR provides the opportunity to assess its temporal changes as part of usual diabetes care. The present data from representative, community-based people with type 2 diabetes show that such serial measurements of uACR can be categorized, using trajectory modeling, into 6 distinct groups with clinical and prognostic significance. The 5 nonnormoalbuminuric groups all had significantly worse glycemic control than individuals with a stable normoalbuminuric trajectory. The 3 relatively small groups that either started from, or had returned to, normoalbuminuria without progression to macroalbuminuria had heterogeneous baseline characteristics but did not exhibit significantly reduced renal function during 4 years of follow-up compared to those in the normoalbuminuric group. The persistent microalbuminuria group comprised individuals who had baseline cardiovascular and metabolic features, and longitudinal renal outcomes, that were adverse relative to the other normoalbuminuria and microalbuminuria groups, but that were not as severe as those of the persistent macroalbuminuria group. Those with persistent microalbuminuria often oscillated between normoalbuminuria and macroalbuminuria on biennial testing over 6 years, but relatively few had progressed to macroalbuminuria at the end of follow-up. The persistent macroalbuminuria group had, consistent with its baseline characteristics, the highest mortality and most rapid progression of renal dysfunction of all 6 groups. These group-specific characteristics could be used to inform management of individuals with type 2 diabetes where prior serial uACR data are available.

In the persistent macroalbuminuria group, the majority had macroalbuminuria at baseline and this had developed by year 2, without any evidence of regression by year 6, in the remainder. Consistent with a range of past studies [29, 30], this group had the highest mortality and the most rapid decline in eGFR from the lowest baseline despite the highest use of RAS-blocking drugs of the 6 groups. Although nonmodifiable risk factors were evident at baseline (male sex and diabetes duration), current smoking, poor glycemic control, systolic blood pressure, and hypertriglyceridemia were also independent predictors of persistent macroalbuminuria group membership. There are scant published data on the efficacy of intensified conventional management of these modifiable risk factors in preventing progression to end-stage kidney disease and death in people with type 2 diabetes and established macroalbuminuria [31-33]. However, the FDS2 was conducted before the widespread availability of the glucagon-like peptide 1 receptor agonists (GLP1-RAs), sodium-glucose cotransporter-2 inhibitors (SGLT2i) and, more recently, finerenone, in Australia, and there is good evidence that these agents offer cardiorenal benefits for people with persistent macroalbuminuria [34-36].

The persistent microalbuminuria group had baseline characteristics that were intermediate between the normoalbuminuria/other microalbuminuria trajectory groups and the persistent macroalbuminuria group, suggesting that these individuals are on the pathway to macroalbuminuria and its adverse prognosis. There is evidence that measures such as smoking cessation, and improved glycemic and blood pressure control, prevent progression to macroalbuminuria and its sequelae in this phenotype [31-33, 37], and the newer agents can provide additional benefits [34-36]. The participants with persistent microalbuminuria also exhibited the greatest within-participant uACR variability over the 6-year follow-up period. It is possible that this phenomenon reflects similar variability in microalbuminuria risk factors such as glycemic control and hypertension, which are themselves associated with a poor prognosis in type 2 diabetes [38, 39]. Nevertheless, the present data provide some evidence that people with type 2 diabetes who have long-term uACR variability merit close follow-up for, and relatively intensive management of, glycemia and other cardiorenal risk factors.

There were 3 trajectory groups (progression, regression, and progression/regression) in which there were distinct overall dynamic changes within the microalbuminuric range over the 6 years. The variables that predicted membership in these groups were also heterogeneous. The closest trajectory group phenotypically to those with persistent microalbuminuria was the progression group, with older age, current smoking, and increased serum triglycerides as shared predictors. The participants in the progression group had no microalbuminuria at baseline but had a relatively short diabetes duration relative to those with a persistent microalbuminuria trajectory (medians 5.0 and 12.0 years, respectively), suggesting that these participants were at an early stage in the progression to persistent microalbuminuria and subsequently to persistent macroalbuminuria.

In the 2 other dynamic microalbuminuria groups (regression and progression/regression), which represented just more than 15% of the total sample, there was early or delayed regression toward normoalbuminuria with remission in the majority of these participants. There are few studies of microalbuminuria remission or regression in type 2 diabetes. In 3 small-scale outpatient clinic-based Japanese studies, 50% [40], 17% [41], and 27% [42] of participants had remission of microalbuminuria over between 3 to 8 years. In the multifactorial interventional Steno study, 30% of participants had remission to normoalbuminuria over 8 years [43]. In all these studies except the most recent [42], the minority of participants were prescribed RAS-blocking drugs during follow-up. Our rate of remission was relatively low but two-thirds of our participants were taking an RAS-blocking drug at baseline. Our 2 groups with microalbuminuria regression were enriched for participants who were not fluent in English and were thus likely recent migrants. It is possible that participation in the present study, including patient and practitioner access to biochemical results, may have increased health care utilization and health literacy in a group of Australians who do not enjoy full access to care [44], with a consequent improvement in microalbuminuria risk factors.

The participants who maintained normoalbuminuria during the 6-year follow-up period were typically younger people with short diabetes duration; relatively low blood pressure, HbA_1c_, and serum triglycerides; and low rates of smoking. They also had a comparatively low baseline prevalence of other microvascular as well as macrovascular complications. Although their annual change in eGFR over 4 years (mean −1.5 mL/min/1.73 m^2^) was within the range of other community-based studies of type 2 diabetes [45], it was not significantly lower than those of the 4 microalbuminuria groups even if the trend analysis suggested an increase across the 6 groups. Since the nonmicroalbuminuric groups had lower baseline eGFRs than those with normoalbuminuria, the percentage annual eGFR change was greater and thus their rates of progression toward eGFR cut-points for chronic kidney disease were faster [28].

The present data from a representative, community-based sample have important and novel clinical implications. First, individuals with type 2 diabetes and persistent macroalbuminuria are highly unlikely to regress to lower categories of urinary albumin excretion and their poor prognosis should prompt consideration of the use of the newer medications (GLP1-RA, SGLT2i, and finerenone) with evidence of cardiorenal benefit [34-36] in addition to optimized conventional risk management. Second, those with year-to-year fluctuations in uACR to and from normoalbuminuria, microalbuminuria, and macroalbuminuria are typically phenotypically on the pathway to persistent macroalbuminuria and warrant similar management to that for people with established macroalbuminuria. Third, individuals with a steady progression from normoalbuminuria to microalbuminuria should be viewed as at an early stage of increased risk of more severe diabetic nephropathy and their management intensified appropriately. Lastly, the 1 in 7 people with type 2 diabetes who regress to consistent normoalbuminuria have a relatively good renal prognosis with conventional care.

The present study had limitations. Observational studies such as the FDS2 can be affected by bias related to study recruitment and retention. Nevertheless, the FDS2 cohort had similar baseline demographic and diabetes-specific characteristics to nonrecruited but eligible people with type 2 diabetes [16], and socioeconomic data from the FDS2 catchment were comparable to national means [25]. We had biennial rather than the recommended annual uACR measurements [10]. However, European studies suggest that up to 50% of people with diabetes managed in a primary care setting do not have an annual uACR [46], while the individual uACR vs time plots shown in Fig. 3, especially those for normoalbuminuria, regression, and persistent macroalbuminuria, would question the value of additional serial uACR data. The present epidemiological analyses should be used in conjunction with individual patient assessment since serial changes in uACR may be affected by factors such as intense exercise, dietary change, intercurrent infections, and changes in as-required and regular medications, in addition to diabetes-specific pathophysiology. The strengths of the present study are the large samples of participants followed for a long period, and its well-characterized participants with type 2 diabetes.

In conclusion, trajectory modeling is increasingly used to identify the phenotypic and prognostic characteristics of subgroups of individuals with a disease such as type 2 diabetes. In the present study, trajectory analysis of biennial uACR measurements over 6 years found 6 robust groups from sustained normoalbuminuria to persistent macroalbuminuria. Their heterogeneous baseline characteristics and outcomes have clinical implications, and could even justify stratification of randomized participants in future clinical trials of new therapies for diabetic nephropathy where albuminuria is a key end point.

## Data Availability

Restrictions apply to the availability of data generated or analyzed during this study to preserve patient confidentiality or because they were used under license. The corresponding author will, on reasonable request, detail the restrictions and any conditions under which access to some of the data may be provided.

## Abbreviations

ABSI: a body shape index
BIC: Bayesian Information Criterion
BMI: body mass index
CCI: Charlson Comorbidity Index
DKD: diabetic kidney disease
DSPN: distal symmetrical polyneuropathy
eGFR: estimated glomerular filtration rate
FDS2: Fremantle Diabetes Study Phase 2
GBTM: group-based trajectory modeling
GLP1-RAs: glucagon-like peptide 1 receptor agonists
HbA_1c_: glycated hemoglobin A_1c_
HDL: high-density lipoprotein
HMDC: Hospital Morbidity Data Collection
PAD: peripheral arterial disease
RAS: renin-angiotensin system
SGLT2is: sodium-glucose cotransporter-2 inhibitors
uACR: urinary albumin:creatinine ratio
uAER: urinary microalbumin excretion rate
WA: Western Australia
